# Turning the World Upside-Down in Cellulose for Improved Culturing and Imaging of Respiratory Challenges within a Human 3D Model

**DOI:** 10.3390/cells8101292

**Published:** 2019-10-21

**Authors:** Viktoria Zaderer, Martin Hermann, Cornelia Lass-Flörl, Wilfried Posch, Doris Wilflingseder

**Affiliations:** 1 Institute of Hygiene and Medical Microbiology, Medical University of Innsbruck, 6020 Innsbruck, Austria; 2 Department of Anesthesiology and Critical Care Medicine, Medical University of Innsbruck, 6020 Innsbruck, Austria

**Keywords:** 3D airway cultures, imaging, longevity

## Abstract

Polarized growth of human-derived respiratory epithelial cells on hydrogel-coated filters offers big advantages concerning detailed experiments with respect to drug screening or host pathogen interactions. Different microscopic approaches, such as confocal analyses and high content screening, help to examine such 3D respiratory samples, resulting in high-resolution pictures and enabling quantitative analyses of high cell numbers. A major problem employing these techniques relates to single-use instead of multiple-use of Transwell filters and difficulties in the digestion of collagen if subsequent analyses are needed. Up to date, cells are seeded in collagen-based matrices to the inner field of Transwell inserts, which makes it impossible to image due to the design of the inserts and hard to perform other analyses since digestion of the collagen matrix also affects Transwell grown cells. To overcome these problems, we optimized culturing conditions for monitoring cell differentiation or repeated dose experiments over a long time period. For this, cells are seeded upside-down to the bottom side of filters within an animal-free cellulose hydrogel. These cells were then grown inverted under static conditions and were differentiated in air-liquid interphase (ALI). Full differentiation of goblet (Normal Human Bronchial Epithelial (NHBE))/Club (small airway epithelia (SAE)) cells and ciliated cells was detected after 12 days in ALI. Inverted cell cultures could then be used for ‘follow-up’ live cell imaging experiments, as well as, flow-cytometric analyses due to easy digestion of the cellulose compared to classical collagen matrices. Additionally, this culture technique also enables easy addition of immune cells, such as dendritic cells (DCs), macrophages, neutrophils, T or B cells alone or in combination, to the inner field of the Transwell to monitor immune cell behavior after repeated respiratory challenge. Our detailed protocol offers the possibility of culturing human primary polarized cells into a fully differentiated, thick epithelium without any animal components over >700 days. Furthermore, this animal-free, inverted system allows investigation of the same inserts, because the complete Transwell can be readily transferred to glass-bottom dishes for live cell imaging analyses and then returned to its original plate for further cultivation.

## 1. Introduction

Live cell imaging is a very important and widely used method in science to analyze and characterize cellular processes, such as proliferation and differentiation. In the case of cultured respiratory epithelium, live cell and high content imaging are also applied for examining mucociliary clearance. Mucociliary clearance provides the first defense against pathogens attaching to the mucous layer, and this mechanism is necessary for full function of the lungs [1,2,3,4]. Different defense molecules ensure the aggregation, trapping, and killing of microbes [5]. Via one-directional beating of cilia, the extracellular fluid (ECF) is moved towards the upper parts of the mammalian airway tree, and the lungs are cleared form inhaled pathogens.

**Figure 1:**
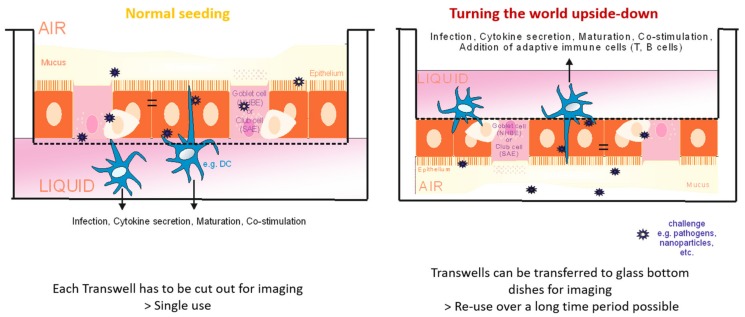
Normal seeding (left) of primary, respiratory epithelial cells (Normal Human Bronchial Epithelial (NHBE), small airway epithelia (SAE)) in cellulose-based GrowDex^®^ does not allow multiple uses of 3D grown tissue, since Transwell inserts have to be cut out or paraffin-embedded for imaging analyses. Imaging is not possible from below due to the thickness of the filter-grown tissue and flipping of normally grown wells is not possible due to the side walls of the Transwell. Upside-down seeding of respiratory cells grown in GrowDex^®^ allows an easy transfer of the Transwell to a glass-bottom dish by lifting the Transwell insert to the other dish under sterile conditions. After-life cell imaging analyses are done, the Transwell insert is transferred back into the original well. Therefore, among many other applications, cell differentiation and mucociliary clearance can be monitored using the same cells grown in an animal-free cellulose hydrogel.

The topic of ‘three-dimensional’ cell culture systems has become more and more important during the last years. Three-dimensional models are now indispensable in research and play an important role, particularly in the development [6] or during developmental aspects [7]. Due to this and the fact that there is a deficiency in reliable in vitro human models, we set up a working and optimized 3D cell culture model for long-term culturing of respiratory epithelial cells [8]. Especially in the case of epithelial cells as entry sites, detailed interactions concerning pathogen dynamics or kinetics can be studied closer to the situation in vivo. For example, interactions of pathogenic fungi as *Aspergillus (A.) fumigatus* and lung epithelial cells have been shown by our group before [8]. This supporting primary research paper successfully used the protocol of 3D primary epithelial/immune cell co-cultures to study pathogen entry sites more realistically but excluded cultivation in a birch-based hydrogel and flipping of the cell cultures upside-down. Thereby, for every condition, a Transwell filter had to be cut out to perform imaging analyses. The idea of turning the respiratory cell world upside-down in cellulose (Figure 1) came to i) avoid sacrificing a Transwell insert for each and every treatment (or also control), ii) monitor differentiation of the respiratory cells from the same Transwell insert, ii) perform mucociliary clearance and repeated exposure experiments, iv) guarantee easy digestion of the cellulose hydrogel for follow-up experiments, and v) guarantee an easy addition of the immune cells to the fully differentiated epithelium for simpler handling. Regarding these facts, we changed the seeding of cells to upside-down flipped Transwell inserts within a birch-based cellulose membrane (Figure 1) so that the cells were growing and differentiating on the other side of the membrane. We compared the methods of normal vs. inverted seeding and collagen vs. GrowDex^®^ matrix with respect to their functionality and handling procedures.

Usually, fluorescent imaging of cells grown on Transwells requires the filter membranes to be cut out and placed onto a slide or multi-well plate with glass-bottom. Other methods, with respect to cells grown on Transwell filters, comprise, for example, visualization of fixed cell sections embedded in paraffin using immunofluorescence or quantitative and qualitative analyses of cell migration by in vitro Transwell assays [9,10,11]. Applications have been described using direct imaging of a polarized cell line placed directly on the membrane support [10]—however, the confocal images lacked quality compared to the images taken in the upside-down approach, and this imaging technique was not feasible using our primary cells grown in a gel. Using the upside-down method (Figure 1), it is possible to transfer the whole insert with cells facing downwards to the glass-bottom dishes for microscopy, so the same samples can be re-used, and live cell imaging can be easily performed without harming the cells. Another positive aspect of seeding cells upside-down to Transwells is that processes, such as proliferation and differentiation, can be observed over a longer time period using the same insert.

In addition to optimizing the seeding of Normal Human Bronchial Epithelial (NHBE) or small airway epithelial (SAE) cells for better imaging, we improved the culture conditions of the cells by replacing rat-tail collagen or Matrigal by a birch-based animal-free cellulose hydrogel (GrowDex^®^, UPM Biomedicals). Using the birch-derived nanofibril gel as an extracellular scaffold illustrated accelerated proliferation, as well as differentiation rates of both respiratory epithelial cell types tested (NHBE, SAE), compared to rat-tail collagen independent on normal or upside-down cell culturing. The application of upside-down seeding within a cellulose hydrogel is not only relevant for basic research questions related to pathogen entry via epithelial/immune cell barriers, but also for repeated dose experiments concerning other respiratory challenges, such as nanoparticles, smoke, and allergens. Therefore, our model offers a broad range of applications in basic and translational research questions as well as regarding pharmaceutical approaches. In particular, for long-term and repeated dose experiments, this protocol provides a valuable tool for a broad target audience.

## 2. Advantages of the Protocol

**Figure 2:**
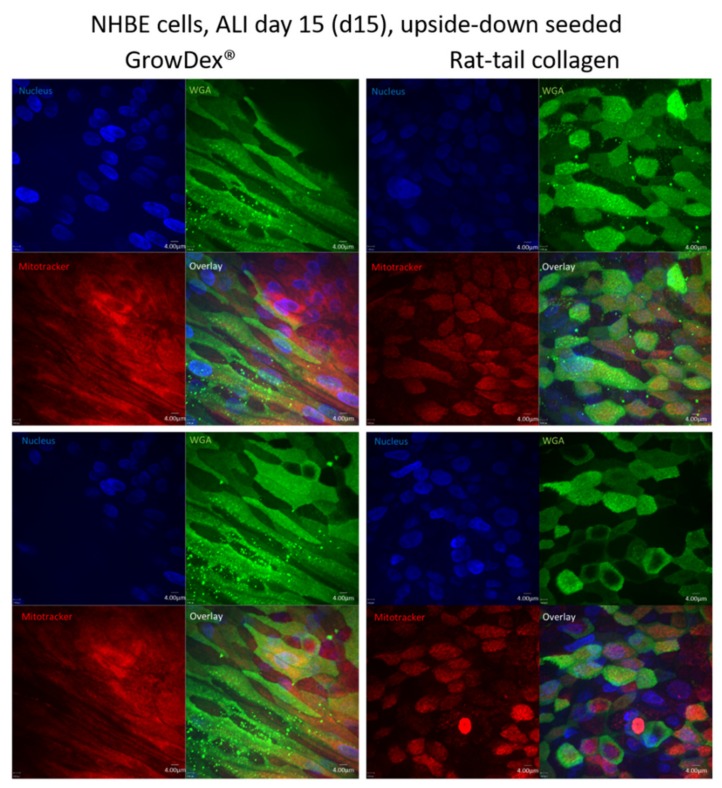
NHBE cells, day 15 (d15) in air-liquid interphase (ALI) and seeded upside-down. Left panel: cells grown on the animal-free, birch-based hydrogel. Right panel: cells grown on rat tail collagen-coated membranes. Images show faster proliferation, due to more cell layers and an increased cell number when the hydrogel was applied. Nuclei were stained using Höchst (blue), cilia using wheat germ agglutinin (green), mitochondria using mitotracker (red). An overlay is illustrated in the bottom right panels. Two representative images from at least 3 independent experiments are depicted for cells cultured upside-down in GrowDex^®^ or rat-tail collagen.

**Figure 3:**
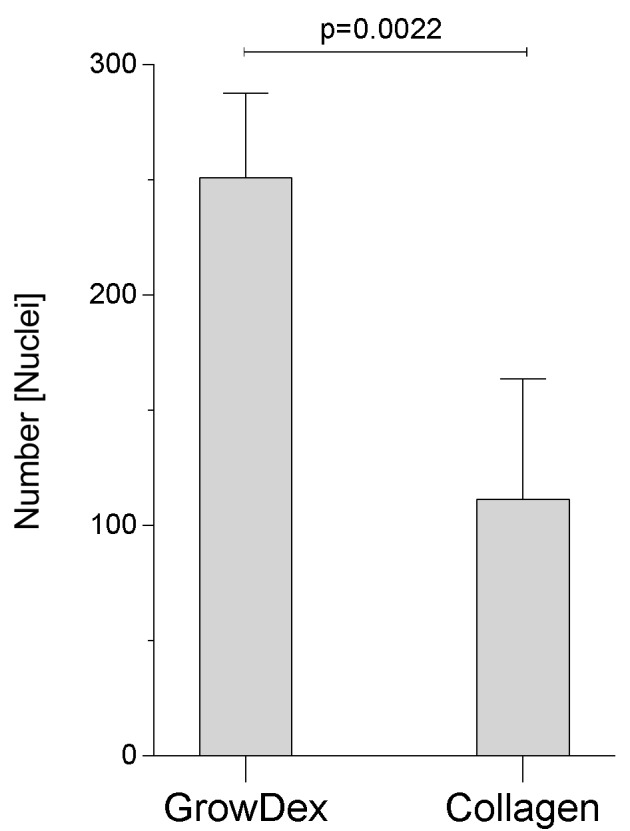
Quantitative analyses of nuclei within GrowDex^®^ and rat-tail collagen. Differentiated cells revealed a significantly higher cell number within the birch-based hydrogel on day 15 post-ALI. Five independent regions of two Transwell inserts were quantified using the Operetta CLS™ HCS, and cells were automatically quantified using the Harmony software.

We applied this protocol working with primary respiratory NHBE and SAE cells from Lonza. NHBE cells originate from the upper part and SAE cells from the lower part of the human airway tree. Typically, the epithelium, grown in rat-tail collagen and under static conditions, was completely differentiated after 21 days in air-liquid interphase (ALI) [8]. Before applying the method of upside-down seeding for improved imaging, we tested growth and differentiation of primary respiratory epithelial cells (NHBE, SAE) within an animal-free cellulose hydrogel and compared these with cells grown in the standardized rat-tail collagen. We found that in GrowDex^®^, respiratory epithelial cells illustrated considerably faster proliferation and differentiation rates compared to rat-tail collagen, and such grown cells were available for further experiments after only two instead of three weeks. Next, we applied both ECM matrices (GrowDex^®^, rat-tail collagen) in combination with the upside-down seeding of primary respiratory epithelial cells (NHBE, SAE). Likewise, normally grown cultures, upside-down seeded cells cultured in birch-based hydrogel, depicted a significantly accelerated proliferation and differentiation, as shown in Figure 2 and Figure 3.

Implementing our new protocol, a completely differentiated and ciliated respiratory epithelium was ready for further experiments after only two weeks under static conditions in air-liquid interphase (ALI) culture. Live cell imaging of upside-down seeded NHBE (Figure 2) and SAE (not shown) cells on day 15 post-ALI illustrated an accelerated proliferation and differentiation rate within the GrowDex^®^ matrix (Figure 2, left) compared to the routinely used rat-tail collagen (Figure 2, right). Cells cultured within the rat-tail collagen matrix exerted similar differentiation properties after three to four weeks in ALI (Figure S1). When quantifying numbers of cells in GrowDex^®^ versus rat-tail collagen on day 15 in ALI, a significantly increased cell number was observed in the birch-based hydrogel as quantitatively analyzed by using the Harmony™ software (Perkin Elmer) (Figure 3). Cilia length, maturation, and numbers of NHBE cells grown in upside-down seeded animal-free, birch-based hydrogel (Supplementary Materials Movie 1) and standard rat-tail collagen (Supplementary Materials Movie 2) were compared. As expected from live cell images (Figure 2), NHBE cells seeded upside-down in GrowDex^®^ showed higher levels of mature cilia, compared to collagen seeded cells, which depicted more immature, growing cilia and only some mature cilia (Supplementary Materials Movie 1 versus Movie 2).

**Figure 4:**
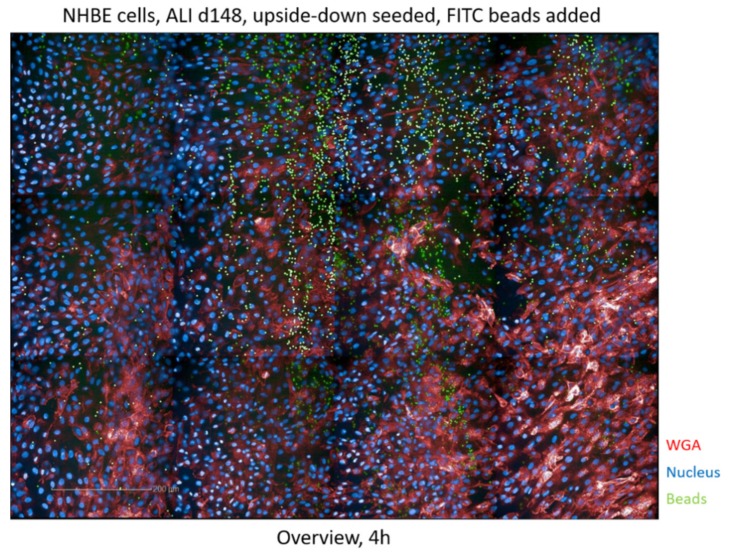
Analysis of mucociliary clearance of NHBE cells cultured in birch-based cellulose hydrogel and upside-down. High content screening of the mucociliary clearance was performed after the addition of fluorescently labeled beads (green) and an overview of the bead distribution is depicted. The experiment was repeated three times in independent experiments.

To investigate whether the upside-down, cellulose-seeded epithelia were functional, mucociliary clearance was checked and analyzed using fluorescently labeled beads and high content screening (HCS) (Figure 4 and Supplementary Materials Movies 3 and 4), as well as live cell imaging (Figure S2).

The overview after 4 h (Figure 4) already illustrates a major distribution of fluorescently labeled beads (green) along ciliated channels, while the minority of beads were unevenly distributed over the tissues. The disorganized arrangement of beads over the well was clearly visible at time point 0 (t0), while after overnight incubation, a pearl chain-like order of the beads was detected (Supplementary Materials Figure S2, t24). The movement of the beads on the epithelium is illustrated in movie 3, while tracking of the beads using the Harmony™ software (Perkin Elmer) is depicted in movie 4. Mucociliary clearance was measured in middle-aged cells on day 48 in ALI (not shown), as well as in older cells on day 148 in ALI (Figure 4, Supplementary Materials Figure S2 and Movies 3 and 4) and the cells did not show any differences in mucociliary clearance independent on the age of the cells.

Overall, upside-down seeding of cells within the birch-based cellulose hydrogel exerted positive effects on proliferation and differentiation of primary respiratory epithelial cells. The advantages of the animal-free cellulose hydrogel comprised a significantly faster differentiation of respiratory epithelial cells also under static conditions, while the upside-down seeding enabled using the same Transwell inserts over time. Therefore, the optimized handling for live cell imaging by seeding the cells upside-down in cellulose makes this protocol ideal for observing processes over a significantly extended period of time up to two years and for multiple exposures. Moreover, mucociliary clearance can be analyzed in a more realistic setting, since the cells are not surrounded by plastic barriers compared to seeding cells in the normal way, which are surrounded by the sides of the Transwell chamber (Figure 1 and Section 4.3.4). Easy addition of immune cells is feasible due to pipetting immune cells into the upper chamber of the insert (Figure 1).

**Figure 5:**
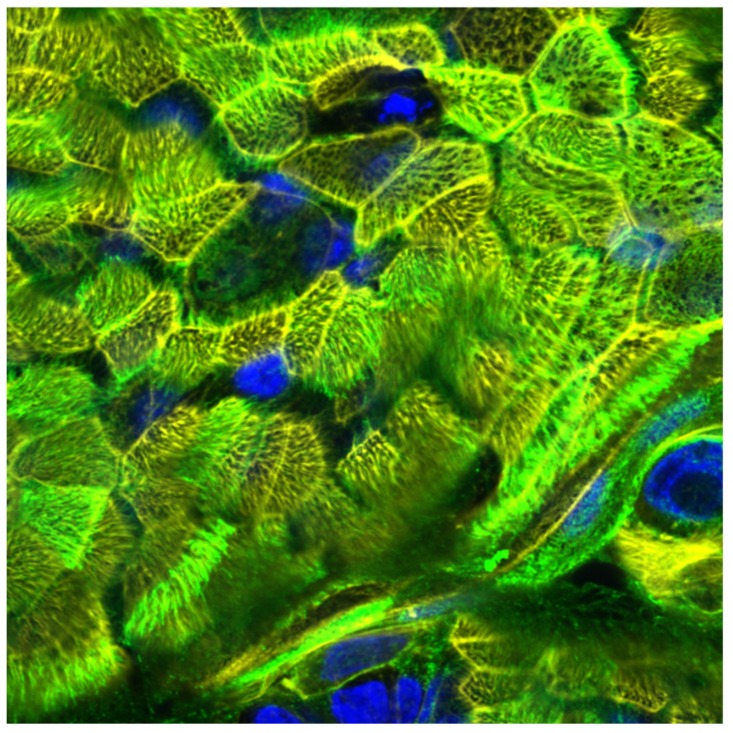
Analysis of epithelial integrity of upside-down cultured NHBE cells on d700 in ALI. Cilia of NHBE cells cultured in GrowDex® for 700 days in ALI and upside-down were stained using WGA-488 (green), cytoskeleton using Phalloidin-Alexa555 (yellow) and nuclei using Höchst (blue).

When we cultured the cells under such conditions, very long culture periods of intact ciliated pseudostratified epithelia up to >700 days were feasible (Figure 5), thereby making this 3D respiratory model best fitted for repeated exposure experiments to monitor novel drugs or compounds over a 2-year period. In our protocol, we illustrated that upside-down, cellulose-seeded primary respiratory cultures show improved rates of proliferation, differentiation, mucociliary clearance, and mucus production, and thus, these 3D models offer an excellent basis for host pathogen interaction studies or purposive complaint-models.

## 3. Limitations

Due to the inverted cultivation of cells, the handling of touching the apical cell surface and, at the same time, reaching the basal chamber of the Transwell, has not been optimized by us so far. Hence, when trans epithelial electrical resistance (TEER) measurements cannot be performed in the normal way, other methods to confirm the integrity of the cultured lung epithelium are necessary. In the case of respiratory epithelial cells, for example, quantifications of mucocilia-ry clearance rates or mitotracker staining or live cell imaging of the actin cytoskeleton provided information about wellbeing, epithelial integrity, and function of the cells. Thus, this possible limitation can be circumvented.

## 4. Detailed Protocol Including !CAUTION and #CRITICAL STEP

### 4.1. Materials

#### 4.1.1. Reagents

- PneumaCultTM-Ex Plus Basal Medium (STEMCELL, Cologne, Germany, cat. no. 05041)
- PneumaCultTM-Ex Plus 50× Supplement (STEMCELL, Cologne, Germany, cat. no. 05042)
- PneumaCultTM- ALI Basal Medium (STEMCELL, Cologne, Germany, cat. no. 05002)
- PneumaCultTM- ALI 10× supplement (STEMCELL, Cologne, Germany, cat. no. 05003)
- PneumaCultTM- ALI 100× Maintenance supplement (STEMCELL, Cologne, Germany, cat. no. 05006)
- Hydrocortisone stock solution (STEMCELL, Cologne, Germany, cat. no. 07925)
- Animal Component-free cell dissociation kit (STEMCELL, Cologne, Germany, cat. no. 05426)
- D-PBS
- Hydrocortisone Solution (STEMCELL, Cologne, Germany, cat. no. 07925)
- Heparin Solution (STEMCELL, Cologne, Germany, cat. no. 07980)
- GrowDex® stock solution (UPM Biochemicals, Helsinki, Finland, cat. no. 100 103 005)
- Mowiol
- WGA 488 (Biotium, Fremont, CA, USA, cat.no. 29022-1)
- Hoechst 33342 (Cell signaling Technology, Frankfurt a. Main, Germany, cat. no. 4082)
- Mitotracker deep red (Life Technologies Austria, Vienna, Austria, cat.no. M22426)
- Recovery Cell culture freezing medium (Gibco™, Life Technologies Austria, Vienna, Austria, cat. no. 12648010)
- Human derived respiratory epithelial primary cells: NHBE: cat#: CC-2540-S, SAEC: cat#: CC-2547-S (Lonza, Cologne, Germany)

#### 4.1.2. Equipment

- Tissue culture flasks, 75 cm2 (T75, Corning Costar, Amsterdam, The Netherlands)
- 6- and 24-well plates (Corning Costar, Amsterdam, The Netherlands)
- Transwell permeable supports, 6,5 mm Insert for 24 well plates (Corning Costar, Amsterdam, The Netherlands)
- Corning Falcon conical centrifuge tubes, 15 mL and 50 mL (Corning Costar, Amsterdam, The Netherlands
- Anatomical tweezers, scissors
- Assorted pipettes (2–20, 20–200 and 100–1000 µL) with sterile, disposable tips
- Centrifuge
- UV-sterilized tape
- Cell culture incubator, 37 °C and 5% CO_2_
- Water bath, 37 °C
- Neubauer chamber
- Vacuum pump, for medium aspiration
- Cryovials
- Cell culture hood
- Inverted microscope for cell culture approaches
- 1 mL serological pipettes
- 10 mL serological pipettes
- 25 mL serological pipettes
- Operetta CLS™ (Perkin Elmer Cellular Technologies Germany GmbH, Hamburg, Germany)
- Confocal microscope (LEICA SP5, Wetzlar, Germany)
- Specimen slides
- Cover slips
- Parafilm

### 4.2. Reagent Setup

Complete PneumaCultTM–Ex Plus Medium: Thaw PneumaCultTM-Ex Plus 50× Supplement at room temperature (15–25 °C). Add 2 mL 50× Supplement and 100 µL Hydrocortisone stock solution to 98 mL of PneumaCult TM-Ex Plus Basal Medium.

PneumaCultTM–ALI Complete Base Medium: Thaw PneumaCultTM-ALI 10× Supplement overnight at 2–8 °C. Mix gently without vortexing and add it to PneumaCultTM–ALI Basal Medium with the concentration 1:10.

PneumaCultTM–ALI Maintenance Medium: Thaw PneumaCultTM–Maintenance Supplement (100×) at room temperature (15–25 °C). Add the following components to 49.15 mL PneumaCultTM–ALI Complete Base medium: 500 µL PneumaCultTM–ALI Maintenance Supplement; 100 µL Heparin Solution; 250 µl Hydrocortisone stock solution.

GrowDex Solution for membrane coating: Pre-warm Complete PneumaCultTM Ex Plus Medium in the water bath at 37 °C. For the preparation of GrowDex^®^ coating solution, the number of Transwell inserts needed has to be calculated first. To seed NHBE/SAE cells upside-down into Transwell inserts, a 0.5% GrowDex^®^ solution was used. Thus, for preparing 1 mL of 0.5% GrowDex^®^, 333 µL of 1.5% stock solution is gently mixed with 567 µl of pre-warmed cell culture medium and 100 µL of cell suspension.

**!CAUTION** Mix components gently by pipetting up and down and try to avoid air bubbles.

### 4.3. Procedure

#CRITICAL STEP During the preparation of reagents and all of the following steps, work under sterile conditions (cell culture hood)!

#### 4.3.1. Seeding of Primary Epithelial Lung Cells to Cell Culture Flask; Expansion Phase

(1) Prepare 100 mL of Complete PneumaCultTM-Ex Plus medium.
(2) Pre-heat 25 mL of the medium to 37 °C (in this case, 25 mL can be transferred to a T75 cell culture flask and pre-heated in the incubator for a minimum of 30 min at 37 °C).
(3) Thaw primary respiratory cells quickly in a water-bath at 37 °C for a maximum of 2 min.

#CRITICAL STEP Only keep primary cells in the water bath until the ice is melted—DO NOT extend the period at 37 °C!

(4) After thawing, immediately transfer cells to pre-warmed growth medium (Complete PneumaCultTM-Ex Plus) in the cell culture flask—between 500,000 and 1 × 10^6^ cells should be seeded to a T75 cell culture flask to guarantee an expansion rate up to 80% of the flask area after 3 days.
(5) Rinse the cryovial with 500 µL of pre-warmed growth medium to make sure all cells are transferred to the T75 flask.
(6) Incubate the flask (lying, not standing) at 37 °C and 5% CO_2_ until the cells expanded to a confluence of about 80%—this takes about 3 days. Using older passages > 4, the expansion phase also takes up to 5 days.
(7) On day 2 of the expansion phase, 10 mL of the medium are exchanged. For this, pre-heat the medium at 37 °C in the water-bath. Aspirate 10 mL of the flask medium by using a serological pipette.

#CRITICAL STEP The cells are attaching to the bottom of the lying flask. For aspirating 10 mL of medium, the flask is put up. Try not to touch the flask edge or scrape cells from the bottom of the flask when aspirating the medium with the serological pipette.

#### 4.3.2. Harvesting of NHBE/SAE Cells from T75 Cell Culture Flasks

After the initial 3 days of the expansion phase, when confluency of 80% is reached, the cells are harvested. For the following steps, when the cells are directly seeded to microporous membranes in Transwells after harvesting, prepare Complete PneumaCult TM-Ex Plus Medium. Also, make sure that tape for fixing the Transwells upside-down is already sterilized by a UV light.

**Figure 6:**
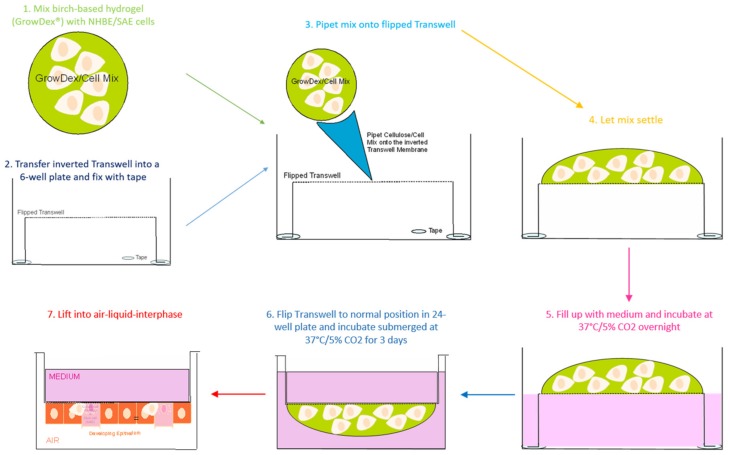
Seeding procedure of primary respiratory cells in cellulose for upside-down cell culture in Transwell chambers. Primary cells (NHBE/SAE) are mixed in GrowDex^®^ as described below and then transferred to an inverted Transwell chamber fixed with tape in a 6-well plate. Cells are allowed to adhere overnight, and the next day, the Transwell chamber is flipped into a 24-Transwell plate. Cells are cultured submerged for 3 days and then transferred into ALI during the differentiation process.

(8) Pre-heat D-PBS (without Mg^2+^ and Ca^2+^) and Complete PneumaCult TM-Ex Plus Medium.
(9) Aspirate the cell culture medium from the upstanding T75 cell culture flask.
(10) Wash the cells with 5 mL pre-warmed D-PBS.
(11) Aspirate the D-PBS and then add 6 mL of ACF Enzymatic dissociation solution.
(12) Add 6 mL of ACF Enzymatic Inhibition solution and mix gently by moving the flask.
(13) Incubate at 37 °C for 7–8 min until cells can be loosened from the bottom by gently tapping to the flask.
(14) Collect the cells in a 15 mL Falcon using a 10 mL serological pipette.
(15) Centrifuge the Falcon at 1400 RPM (350× *g*) for 5 min.
(16) Aspirate the supernatant and re-suspend in pre-warmed Complete PneumaCult TM-Ex Plus Medium.
(17) Count the cells using a Neubauer Chamber.
(18) In the next step, calculate the cell numbers needed for the number of Transwells that are prepared.
(19) By using the first three passages of NHBE and SAE cells, which can be frozen without losing any of the cellular features (see Freezing of non-seeded cells), between 10–13 × 10^6^ cells are harvested from the T75 cell culture flask after the first expansion phase. Then, 1 × 10^6^ cells are added to a new T75 cell culture flask and further expanded (repeat steps from 4) on), while the rest is frozen (see 20) to 24)) or seeded on Transwell filters (see Section 4.3.4 Upside-down seeding of NHBE/SAE cells (Figure 6)).

#### 4.3.3. Freezing of Non-Seeded Cells

(20) Cells that are not immediately used for further culture can be frozen up to a passage of 4, without losing any features (ciliogenesis, proliferative capacities).
(21) Cryovials are prepared with correct labeling.
(22) The calculated cell number seeded on Transwell membranes or further expanded in a T75 flask is separated from cells to be frozen, which are then spun at 1400 RPM (350× *g*).
(23) The supernatant is aspirated, and the pellet is directly re-suspended using 500 µL Recovery cell culture freezing medium per million cells.
(24) Cells are transferred to cryovials before being transmitted into a freezing container for cell preservation to ensure reproducible controlled-rate freezing.

#### 4.3.4. Upside-Down Seeding of NHBE/SAE Cells

(25) Per Transwell insert, 1 × 10^5^ cells are seeded. The number of used Transwells has to be determined first to calculate cell numbers mixed with the GrowDex^®^ solution.
(26) Per upside-down seeded Transwell, 100 µL of 0.5% GrowDex^®^ solution containing cells is used. To get this concentration, 333 µL of GrowDex^®^ stock solution (1.5%) is mixed with 567 µL diluent (Complete PneumaCult TM-Ex Plus Basal Medium) and 100 µL cell suspension.
(27) After the last centrifugation step (see 18), when the calculated cell numbers for seeding are pelleted, the cell pellet is directly solved in pre-heated (37 °C) Complete PneumaCult TM-Ex Plus Basal Medium.

For example: when 10 Transwells are needed, 1 × 10^6^ cells are re-suspended in 100 µL of medium, cells are added to 333 µL GrowDex^®^ stock solution (1.5%) and 567 µL diluent, and on each Transwell, 100 µL of 0.5% GrowDex^®^ containing 1 × 10^5^ cells are seeded.

(28) The cell-containing GrowDex^®^ solution is incubated at 37 °C until Transwell inserts are readily prepared for seeding.
(29) To seed the cells upside-down, the Transwells are fixed in 6-well plates upside-down.

#CRITICAL STEP To fix Transwells in 6-well plates, make sure you are working sterile!

When Transwell inserts are flipped, they are fixed in the 6-well plates by taping the overlaying edge to the bottom of the plastic plates. Make sure you are not touching the membranes facing upwards in this step.

(30) When all Transwells are fixed in 6-well plates in the upside-down position, 100 µL of 0.5% GrowDex^®^ solution/cell mixture is added to the membranes.
(31) When cells are freshly seeded to Transwells, they, again, are undergoing an expansion phase during the first 3–4 days. For this, cells have to be cultured under submerged conditions. During the first hours of overnight incubation of upside-down seeded cells, it is not a problem to cover cells with medium from the apical side because there is medium added to the GrowDex^®^ solution as diluent.

#CRITICAL STEP To cover the cells from the basolateral side is the most critical step!

Chambers containing the fixed Transwells have to be flooded with pre-heated Complete PneumaCult TM-Ex Plus Basal Medium. The most important step here is to avoid air bubbles inside the flipped Transwells. Air bubbles attaching to the membrane prevent cells from sticking to the membrane from the upper side.

(32) In the next step, wrap parafilm around the plate so that the lid does not touch the GrowDex/cell drop on top of the membranes
(33) After an overnight incubation at 37 °C and 5% CO_2_, flip the Transwells back to the normal position and insert them to 24-well plates.
(34) Add 500 µL pre-warmed Complete PneumaCult TM-Ex Plus Basal Medium to the basal chamber of the wells, which will then be the apical layer of the cells to be put in the air phase. Medium is also added to the inside of the Transwells to guarantee submerged conditions (liquid–liquid phase conditions).
(35) Change the medium every second day.
(36) Shift cells to ALI when confluency of 80% is reached, which is normally after about 3 days.

#### 4.3.5. Shift to ALI (Air Liquid Interphase)

(37) For the shift to the ALI state, prepare PneumaCultTM-ALI Basal Medium and PneumaCultTM- ALI Maintenance medium.
(38) For every upside-down seeded Transwell, 250 µL of medium is needed.
(39) Pre-heat the Maintenance medium at 37 °C.
(40) Aspirate the medium at both sides of the membrane and shift the Transwells into new wells.
(41) **!CAUTION** The ALI-Maintenance medium is, from now on, only added to the basolateral side of the cells. So in the case of the upside-down seeded cells, the medium has to be added inside the Transwell and not into the 24-well plate directly under standard culture conditions.
(42) Change the medium every second day after 21 days in ALI culture, and on collagen, epithelial cells are completely differentiated independent on the seeding type (normal or upside-down) and are ready for further experiments. In contrast, cells grown in GrowDex^®^ can be used from day 14 and on in ALI.
(43) **!CAUTION** If culturing cells over a long period of time, the mucous has to be carefully aspirated once a week using a 100 µL tip.

#### 4.3.6. Preparing Cells for Live Cell Imaging

To monitor upside-down and standard seeded respiratory cells for their differentiation and survival, a live cell imaging approach using Hoechst 33342 (nuclei), WGA-488 (surface structures/cilia), and mitotracker in deep red (mitochondria) was chosen.

(44) For preparing the master mix, all compounds were diluted in D-PBS. Small amounts of Master mixes (50–100 µL/well) are added to the bottom of a glass-bottom dish at concentrations found in Table 1.

(45) Optional: To guarantee improved staining of the cells, and in case the mucous is not of interest for imaging, carefully aspirate the mucus layer on the apical side of the cells.
(46) Transfer the Transwell directly to the Master mix in the glass-bottom dish.
(47) Imaging can be immediately started, but fluorescence intensities increase with time. The advantage of seeding cells upside-down, concerning live cell imaging approaches, is that the complete Transwell can be transferred to a sterile 6- or 12-well glass-bottom plate without cutting the membrane. Upside-down seeded Transwells can—after the imaging period—be taken back into the culture without any harm to the cells.

## 5. Conclusions

Upside-down seeding of primary respiratory cells within a cellulose hydrogel provides an efficient method for monitoring cellular proliferation, survival, and differentiation over a period of two years. The optimized culture within the birch-based hydrogel on an inverted Transwell filter makes this protocol perfect for analyzing the same cells under live conditions and for multiple exposures. Moreover, using the upside-down seeding culture, mucociliary clearance can be analyzed in a more realistic way since the cells are not surrounded by plastic barriers from the Transwell chamber, as is the case when seeding cells are in the normal setting. In summary, here, we illustrated that cellulose-grown, upside-down cultures show an accelerated differentiation compared to culture in rat-tail collagen, and in addition, are fully functional, respecting mucociliary clearance and mucus production. Such cultured 3D models offer a very good basis for host-pathogen interaction studies or purposive complaint-models. Also, the mucociliary clearance or, in general, the ciliary beating, can be analyzed quantitatively using this model and, for example, the Harmony™ software (Perkin Elmer).

In addition, the easy addition of immune cells will be feasible due to transferring immune cells simply into the upper chamber of the insert and applying the stimulus directly into the glass-bottom dish to monitor real-time immune cell behavior upon activation. Omitting all animal components in the system avoids unspecific cross-species reactivity and contributes to the replacement of animals in basic research. Moreover, the cellulose hydrogel can be easily digested by using cellulose, which facilitates the detailed characterization of cells by subsequent analyses, such as multi-parameter flow cytometry. In summary, culturing cells under such conditions greatly facilitates not only live cell analyses of the same cells in repeated dose experiments to monitor novel drugs and compounds, and thus to mimic chronic stimulation of the epithelial barrier, but also facilitates the addition of relevant immune cells and subsequent experimental procedures.

## Figures and Tables

**Table 1:** Concentration range of Hoechst, WGA, and mitotracker.

Antibody	Final Concentration
Hoechst 33342	2 µg/mL
WGA-488	5 µg/mL
Mitotracker in far red	100–500 nM

## Supplementary Materials

The following are available online at https://www.mdpi.com/2073-4409/8/10/1292/s1: Movie 1, Cilia differentiation of NHBE cells, day 15 in ALI, and seeded upside-down in rat-tail collagen. Upside-down cultured NHBE cells cultured in rat-tail collagen showed a high amount of immature cilia on day 15 (d15) in ALI; Movie 2, Cilia differentiation of NHBE cells, day 15 in ALI, and seeded upside-down in GrowDex^®^. Upside-down cultured NHBE cells cultured in GrowDex^®^ hydrogel showed a high differentiation of mature cilia all over the epithelium on day 15 in ALI; Movie 3., Fluorescently labeled beads are transported via the upside-down seeded epithelia of d148 NHBE cells. Fluorescently labeled beads are added to NHBE cells stained with Hoechst (blue) and WGA (red) and monitored using HCS; Movie 4, Tracking of beads using the Harmony™ software (Perkin Elmer) after HCS. The mucociliary clearance was monitored using a fully automated setup within the Harmony™ software. 10 µL of microbeads were placed in the glass-bottom dish, and then the upside-down Transwell was added. Pictures were taken as fast as possible in order to reconstitute movies showing the movement of the microbeads. The microbeads movement was tracked using the Harmony™ software for calculating velocities of beads clearance. Figure S1, NHBE cells, day 25 in ALI and rat-tail collagen seeded upside-down. Cells were grown on rat tail collagen coated membranes and after 25 days, such cultured cells illustrate similar cell morphology and differentiation as NHBE cells cultured in birch-based hydrogel after 15 days (Figure S1, left). Nuclei were stained using Höchst (blue), cilia using wheat germ agglutinin (green), mitochondria using mitotracker (magenta). An overlay is illustrated at the bottom right panels. Independent experiments were performed at least three times. Figure S2, Time-dependent arrangement of fluorescently labeled beads on NHBE cells cultured in birch-based hydrogel and upside-down. Addition of fluorescently labeled beads to upside-down seeded NHBE cells, time 0 (t0), illustrates an uneven distribution of the beads, while beads are arranged in a pearl-chain-like manner after overnight incubation (t24). Two independent representative images are depicted.

## References

[1] Gamm U.A., Huang B.K., Mis E.K., Khokha M.K., Choma M.A. (2017). Visualization and quantification of injury to the ciliated epithelium using quantitative flow imaging and speckle variance optical coherence tomography. Sci. Rep..

[2] Chateau S., D’Ortona U., Poncet S., Favier J. (2018). Transport and mixing induced by beating cilia in human airways. Front. Physiol..

[3] Puchelle E., Zahm J.-M., Tournier J.-M., Coraux C. (2006). Airway epithelial repair, regeneration, and remodeling after injury in chronic obstructive pulmonary disease. Proc. Am. Thorac. Soc..

[4] Thompson A., Robbins R., Romberger D., Sisson J., Spurzem J., Teschler H., Rennard S. (1995). Immunological functions of the pulmonary epithelium. Eur. Respir. J..

[5] A Whitsett J., Alenghat T. (2015). Respiratory epithelial cells orchestrate pulmonary innate immunity. Nat. Immunol..

[6] Langhans S.A. (2018). Three-dimensional in vitro cell culture models in drug discovery and drug repositioning. Front. Pharmacol..

[7] Chen Y.-W., Huang S.X., De Carvalho A.L.R.T., Ho S.-H., Islam M.N., Volpi S., Notarangelo L.D., Ciancanelli M., Casanova J.-L., Bhattacharya J. (2017). A three-dimensional model of human lung development and disease from pluripotent stem cells. Nature.

[8] Chandorkar P., Posch W., Zaderer V., Blatzer M., Steger M., Ammann C.G., Binder U., Hermann M., Hörtnagl P., Lass-Flörl C. (2017). Fast-track development of an in vitro 3D lung/immune cell model to study Aspergillus infections. Sci. Rep..

[9] Rayner R.E., Makena P., Prasad G.L., Cormet-Boyaka E. (2019). Optimization of normal human bronchial epithelial (NHBE) cell 3D cultures for in vitro lung model studies. Sci. Rep..

[10] Bergeron A., Rossi A. Imaging of Cellular Fluorescence on Corning’s Transwell® Permeable Supports. https://www.corning.com/catalog/cls/documents/application-notes/CLS-AN-521-A4.pdf.

[11] Pijuan J., Barceló C., Moreno D.F., Maiques O., Sisó P., Marti R.M., Macià A., Panosa A. (2019). In vitro Cell Migration, Invasion, and Adhesion Assays: From Cell Imaging to Data Analysis. Front. Cell Dev. Boil..

