# Supplementary material for: Turning the World Upside-Down in Cellulose for Improved Culturing and Imaging of Respiratory Challenges within a Human 3D Model
Source: Cells. 2019 Oct 21;8(10):1292. doi: 10.3390/cells8101292 (PMC6830077; doi:10.3390/cells8101292)

Supplementary Figures

Suppl. Figure 1. NHBE cells, day 25 in ALI and rat-tail collagen seeded upside-down. Cells were grown on rat tail collagen coated membranes and after 25 days, such cultured cells illustrate similar cell morphology and differentiation as NHBE cells cultured in birch-based hydrogel after 15 days (Fig. 1, left). Nuclei were stained using Höchst (blue), cilia using wheat germ agglutinin (green), mitochondria using mitotracker (magenta). An overlay is illustrated at the bottom right panels. Independent experiments were performed at least three times.

NHBE cells, ALI d25, upside-down seeded

Rat-tail collagen

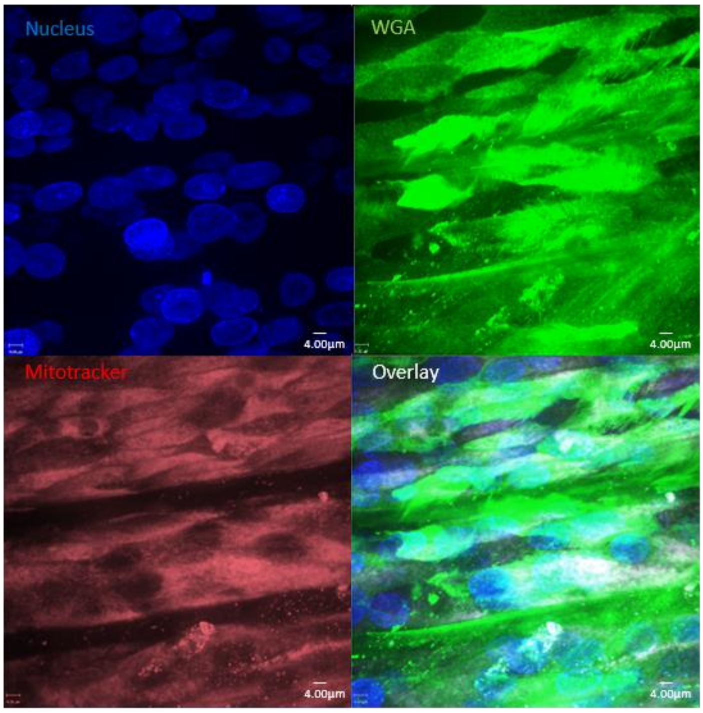

Suppl. Figure 2. Time-dependent arrangement of fluorescently labeled beads on NHBE cells cultured in birch-based hydrogel and upside-down. Addition of fluorescently labeled beads to upside-down seeded NHBE cells, time 0 (t0), illustrates an uneven distribution of the beads, while beads are arranged in a pearl-chain-like manner after overnight incubation (t24). Two independent representative images are depicted.

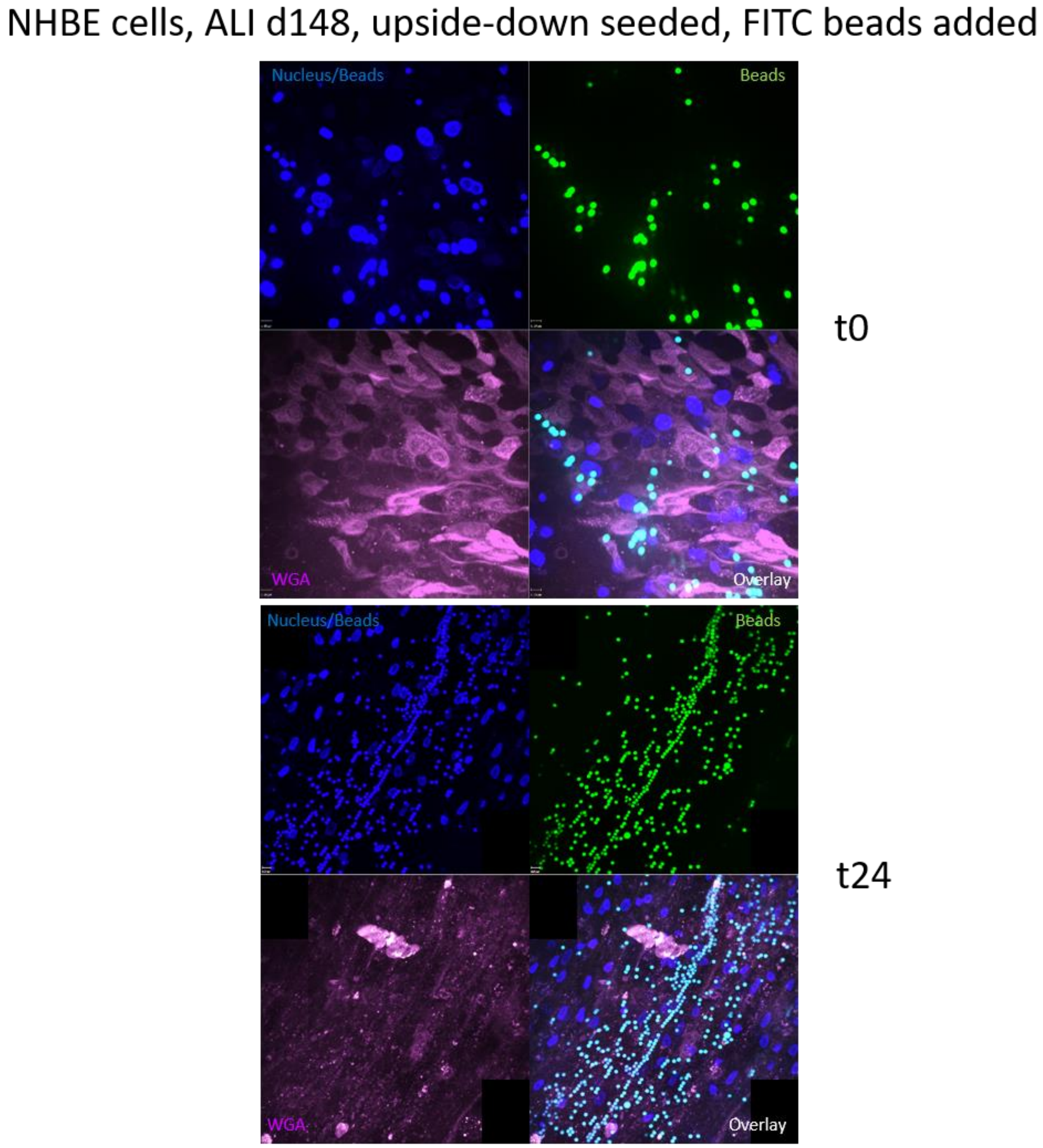

Supplement: Supplementary file 1 [file cells-08-01292-s001.zip › Supplementary Figures.pdf]
